# Ground-based passive generation of Solar Particle Event spectra: Planning and manufacturing of a 3D-printed modulator

**DOI:** 10.1016/j.zemedi.2023.10.002

**Published:** 2023-11-07

**Authors:** Tabea Pfuhl, Uli Weber, Felix Horst, Marco Durante, Christoph Schuy

**Affiliations:** a GSI Helmholtzzentrum für Schwerionenforschung GmbH, Darmstadt, Germany; b OncoRay - Helmholtz-Zentrum Dresden-Rossendorf, Dresden, Germany1 Present address.^1^; c Technische Universität Darmstadt, Institut für Physik Kondensierter Materie, Darmstadt, Germany

**Keywords:** Solar particle event, SPE, Modulation, 3D printed modulator, Energy spectra, Space radiation

## Abstract

The generation of space radiation on Earth is essential to study and predict the effects of radiation on space travelers, electronics, or materials during future long-term space missions. Next to the heavy ions of the galactic cosmic rays, solar particle events play a major role concerning the radiation risk in space, which consist of intermediate-energy protons with broad spectra and energies up to a few hundred MeV. This work describes an approach for the ground-based generation of solar particle events. As a proof of principle, a passive beam modulator with a specific funnel-shaped periodic structure was designed and is used to convert a monoenergetic proton beam into a spectral proton energy distribution, mimicking a solar particle event from August 1972, which is known as one of the strongest recorded SPE events. The required proton beam of 220 MeV can be generated at many existing particle accelerators at research or particle therapy facilities. The planning, manufacturing and testing of the modulator is described step by step. Its correct manufacturing and the characteristics of the solar particle event simulator are tested experimentally and by means of Monte Carlo simulations. Future modulators will follow the same concept with minor adjustments such as a larger lateral extension. As of now, the presented beam modulator is available to the research community to conduct experiments at GSI for exposure under solar particle event conditions. In addition, researchers can use and apply the described concept to design and print their individualized modulator to reproduce any desired solar particle event spectrum or request the presented modulator geometry from the authors.

## 1 Introduction

Hazardous effects induced by space radiation are arguably one of the main show-stoppers for manned space exploration outside the protection zone of Earth’s magnetic field and atmosphere [1], [2]. Astronauts and mission-critical electronics alike, constantly interact with highly energetic heavy ions of the galactic cosmic rays (GCR) [3] as well as sporadic, high-intensity bursts of mainly intermediate energy protons produced by solar particle events (SPE) [4]. Even though the relatively constant GCR exposure is normally considered as the higher risk for long-term space missions, the hazard that could come from SPE bursts is difficult to assess because reliable electronic records have only been available for less than 100 years. There is some indication that there have been extremely strong SPE events in the longer past, e.g. the Carrington (1859) or the much earlier Miyake events [5]. In this regard, the relevance of the research on SPEs is also very high. Acute or constant and long-term exposure to the space radiation environment can induce several cancerous and non-cancerous effects in astronauts and additionally poses a serious problem for the reliability of essential spacecraft electronics [6]. To study, risk-asses and mitigate these deleterious radiation effects, experiments with space-like radiation are essential for the safe, manned exploration of the solar system. However, conducting experiments directly in space is not always technically feasible and extremely cost-prohibitive, therefore ground-based high-energy particle accelerators are a crucial tool to further study the impact of space radiation.

Classically, biological samples or electronic devices under testing are exposed to monoenergetic ions (in case of GCR) or protons (in case of SPE) provided by particle accelerators as a substitute for the space radiation environment to study and extrapolate radiation effects prevalent in long-term missions. However, this approach leads to a significant reduction in the complexity of the produced radiation field and, therefore, can not be used to study synergistic effects induced by different radiation qualities simultaneously hitting the target in close proximity [7].

To combat these limitations, NASA’s Space Radiation Laboratory (NSRL) developed and implemented advanced space radiation simulators to more closely mimic the radiation field complexity of both GCR [8] and SPEs [9] exposures. The GCR simulation sequentially exposes a target to predefined fluences of five different ion species at several energies each, as well as additional exposures to hydrogen and helium ions in combination with a passive energy degrader. Additionally, NSRL offers two simulated reference SPE energy spectra modeled after the events observed in 1972 and 1989. While running the SPE simulation, the target is consecutively irradiated with a series of proton beams (with a fixed synchrotron energy) that are differently degraded by a set of range shifter plates made of polyethylene with different thicknesses. During this irradiation sequence, the beam intensity is modulated, too: the partial irradiations with thick absorber plates use much higher fluences than for thin absorber plates. The different absorber thicknesses and fluences in the sequence are optimized for the best possible fit to the given SPE spectra in the range between 50 and 150 MeV. This technique closely resembles the creation of a spread-out Bragg peak as used in particle therapy, however, the energy spectra and the depth dose profiles have a completely different (inverse) character.

To offer high-quality space radiation simulation for the European space radiation research community, the GSI Helmholtzzentrum für Schwerionenforschung GmbH (GSI), supported by the European Space Agency (ESA), is currently developing advanced space radiation simulators following a different concept. GCR simulations will employ a hybrid active–passive approach [10] using several monoenergetic ${}^{56}$Fe beams interacting with different complex, passive beam modulators to instantaneously mimic a GCR reference field.

The SPE modulator presented in this work is a simpler development than the GCR simulator, since it has to provide the energy spectrum for one particle species (protons) only, which neither can produce lighter projectile fragments. However, this project serves as a good preparation for the more complex passive GCR modulator. The concepts for the optimization of the spectra or for the design and printing of the modulators could be partly adopted. Conceptional differences between SPE modulator and GCR modulator are explained in the discussion section.

The GCR simulator work from 2020 [10] already outlines the design of the SPE modulator and shows some preliminary MC simulations. However, the present work describes in detail the design process, production and validation of a complex passive modulator capable of reproducing the August 1972 SPE spectrum [11], which was one of the most dangerous recorded events and a commonly used reference SPE in literature. Although the simulated spectrum in [9] is limited to 150 MeV, in this work the maximum energy of therapy facilities (220 MeV) was chosen, to adapt more accurately to the 1972 spectrum and to provide also the slight dose distribution of the particles from 150–200 MeV, which are stopping in the depth range from 16 to 25 cm in water.

The modulator is a 3D-printed device in steel with a periodic filigree structure. Instead of irradiating long sequences using different absorber plate configurations (as for the NSRL concept), the presented modulator can instantaneously convert a monoenergetic beam into the desired spectrum.

It should be noted that the concepts for the SPE modulator were adapted from the patient-specific 3D-printed range modulators for particle therapy and FLASH ion-beam application [12], [13], [14], which were developed in the same working group. The main difference is, that the SPE modulator is not optimized for a specific depth-dose distribution but for an energy spectrum.

One important application of the SPE simulator in the near future will be the efficient (fast) irradiation and testing of electronic modules for satellites and space missions within the EU-funded HEARTS [15] project.

## 2 Methods

The shape of the proton’s kinetic energy spectrum and the intensity of SPEs varies from event to event [16]. For the SPE modulator described in this work, we selected the SPE spectrum measured in August 1972 [17], extracted from Fig. 3 in [16] because this event was particularly strong and, thus, especially useful for conservative risk assessments of space radiation and additionally as a proof of principle. However, in general, the described procedure can be applied to any available SPE spectrum within the limits of the modulator manufacturing process. The presented modulator was manufactured from steel with a density of 7.9 g/cm^3^ in the 3D-printing technique (see below, manufacturing of the modulator). The modulator design, manufacturing, and testing are described in detail in the following subsection.

### 2.1 Modulator development

**Figure 1:**
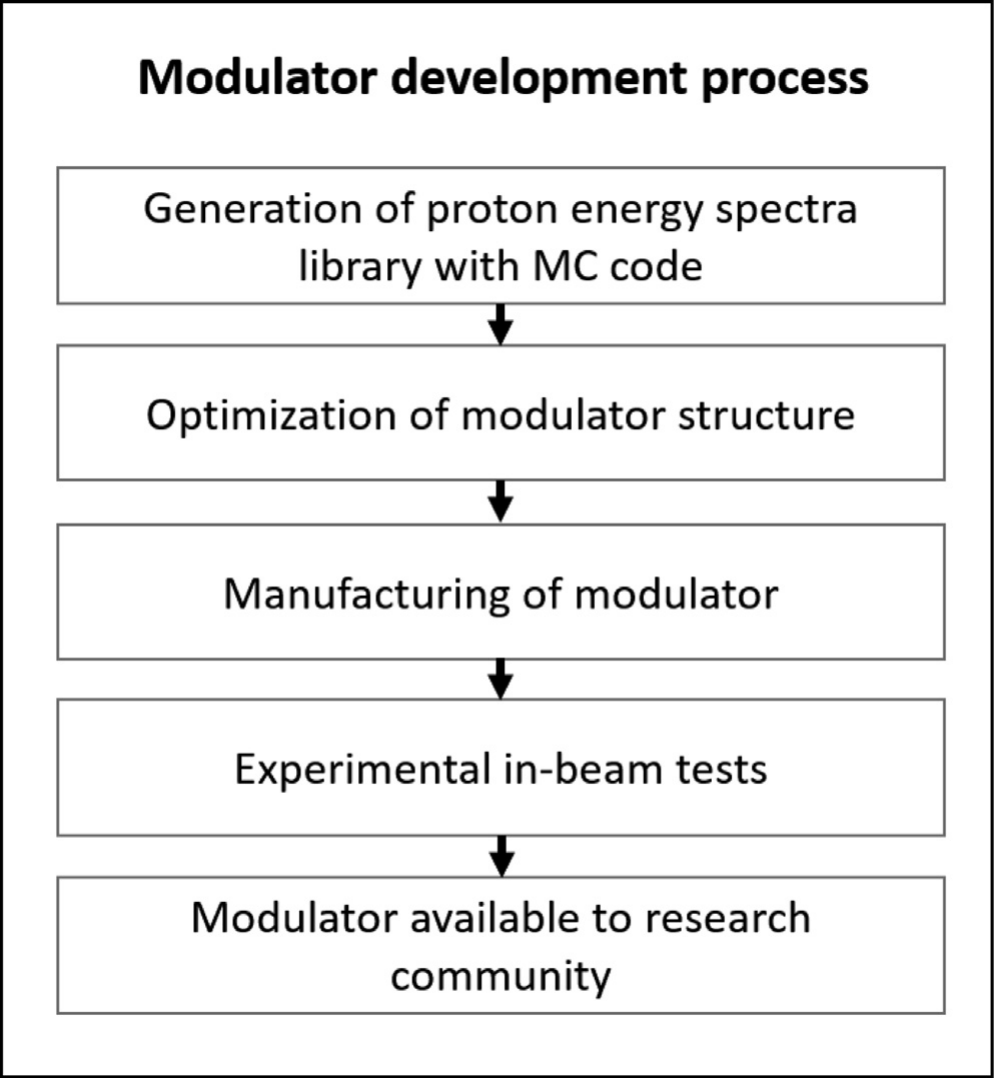
Process flow chart for modulator development from the first planning to its broad availability to the research community.

**Figure 2:**
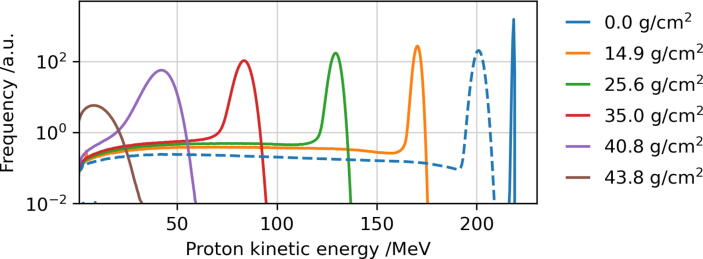
Kinetic energy spectra of a 220 MeV proton beam passing selected iron material thicknesses as indicated by the areal density in the legend (frequency given in arbitrary units). The solid lines visualize the energy spectra simulated with FLUKA ${\mathrm{Spc}}_{i,j}$ and the dashed line shows the broadened (by the LN300 foam) energy spectrum ${\mathrm{SpcF}}_{i,j}$ obtained by Eq. (2), exemplary at the entrance surface of the modulator (t = 0 g/cm^2^).
• **Optimization of the modulating cell structure:**
  In order to optimize the shape of the structure of the SPE modulator, first a series of weights $w_{i}$ has to be determined in a way, that the spatial convolution of the spectra ${\mathrm{SpcF}}_{i,j}$ with the weight $w_{i}$ yields the desired energy spectra ${\mathrm{SPE}}_{j}$ for the 1972 event. The discretization (energy binning) of ${\mathrm{SPE}}_{j}$ is the same as for the simulated spectra ${\mathrm{Spc}}_{j}$ and ${\mathrm{SpcF}}_{j}$. MATLAB (R2019) was used to perform the following $\chi^{2}$- minimization:
  (3) $$\chi^{2}(w_{0},\ldots ,w_{1700})=\underset{i,j}{\sum }{\left[\left({\mathrm{SpcF}}_{i,j}\times w_{i}\right)-{\mathrm{SPE}}_{j}\right]}^{2}$$
  For the presented modulator the integration limits were chosen as i=0,…,1700 and j=80,…,406, corresponding to the areal densities in iron from 0 to 36.4 g/cm^2^ and to the energy range from 40 to 203 MeV, respectively. The following method was applied to avoid oscillations during optimization with the very fine binning grid of $w_{i}$: Only a subset of the weights ${\overset{\sim }{w}}_{i}$ was directly iterated during the optimization process by MATLAB, whereas the remaining weights were implicitly optimized in the optimization routine by interpolation between the subset ${\overset{\sim }{w}}_{i}$. The interpolated full set of weights $w_{i}$, the subset ${\overset{\sim }{w}}_{i}$ and the resulting SPE spectrum of the optimization can be seen in Fig. 6. With the optimized weights $w_{i}$ the full, optimized SPE spectrum can be calculated as:
  (4) $${\mathrm{SPE}}_{\mathrm{opt},j}=\underset{i=0,\ldots ,1700}{\sum }{\mathrm{SpcF}}_{i,j}\times w_{i}$$
  After optimization, the weights must be normalized to $\sum w_{i}=1$. In a next step the weights have to be transferred into the design of the cells of the modulator array with period λ. The common concept is to divide the volume of the cell (with square base $A_{q}=\lambda^{2}$) into many very fine layers $A_{i}$ perpendicular to the beam and located in a depth $t_{i}=\mathrm{dt}\cdot i$ . The concept of the layer subdivision is depicted in a very simplified form in Fig. 3. The areas of the layers are assigned to the weights $w_{i}$ as follows:
  (5) $$\Delta A_{i}=A_{i}-A_{i+1}=A_{q}\cdot w_{i};\Delta A_{0}=A_{q}\cdot w_{0};A_{i_{\mathrm{top}}}=A_{q}\cdot w_{i_{\mathrm{top}}}$$
  with $\Delta A_{i}$ as the effective area proportional to the optimized weights $w_{i}$. The depth $t_{0}$ corresponds to zero thickness in steel, meaning this is the area of the opening $\Delta A_{0}$ (hole) at the bottom of the cell, $A_{i_{\mathrm{top}}}$ denotes the area at the top of the cell, where the thickness is given by $i_{\mathrm{top}}\cdot \mathrm{dt}$ = 36.4 g/cm^2^ for the presented modulator. Normally, the layers are divided much finer than shown in Fig. 3. If the layer thicknesses are fine, then the steps can be replaced by a shape with a smooth gradient, as depicted in Fig. 3b. Basically, the 2D-shape of $A_{i}$ can be arbitrary, the only condition is that $A_{i}$ always fully covers $A_{i+1}$. We opted for a square with full side length λ of the cell and a circular opening $r_{i}$, leading to a circular funnel for the whole set of $A_{i}$. The circle edge intersects with the square border of the cell above a certain height. This has to be considered by the following formula defining the area $A_{i}$ as a function of the radius (see also Fig. 4).
  (6) $$A_{q}-A_{i}(r_{i})=\left\{\begin{array}{cc} \pi r_{i}^{2}, & :r_{i}⩽\frac{\lambda }{2}\\ \sqrt{{\left(2\lambda r_{i}\right)}^{2}-\lambda^{4}}+\left[\pi -4\;\arccos(\lambda /2r_{i})\right]r_{i}^{2}, & :\frac{\lambda }{2}<r_{i}⩽\frac{\lambda }{\sqrt{2}} \end{array}\right)$$

The working principle of the SPE modulator can be explained as follows. The modulator has a 2D-periodic array of cells each with a square base. All cells contain the same structure that enables the desired modulation behavior. The shape of a single cell can be understood as a stack of many different layers orthogonal to the beam direction. Thus, if this modulator is irradiated with a proton beam, depending on the location a particle passes through the periodic shape, it penetrates a specific material thickness. Primary protons are slowed down in the material and secondary protons with lower energies than the primaries are produced in nuclear reactions. Downstream of the SPE modulator, a certain proton energy spectrum resulting from the superposition of many particles that passed different material thicknesses will be found. For the modulator presented in this work, the resulting energy spectrum adapts to the 1972 SPE spectrum in the energy range from 40 to 200 MeV. These two limits were chosen because, on the one hand, dose contributions beyond 200 MeV in the 1972 spectrum are negligible and, on the other hand, energies below 40 MeV (corresponding to approx. 1.5 cm range in H_2_O) are easily shielded by the hull of a spacecraft. The distinct structure and the resulting inhomogeneous scattering of the SPE modulator leads to an inhomogeneous fluence and dose distribution directly behind the modulator. However, due to the multiple scattering and the fine structure, the lateral inhomogeneities are blurred in a distance of typically ≈10 cm behind the modulator (see results section). Therefore, this distance is suitable to irradiate a target such as a cell culture flask with a smooth lateral dose and fluence distribution. This blur-out effect is well known from modulators in particle therapy [18], [19]. The process to produce a modulator from planning to availability to end-users in the research community is visualized in Fig. 1 and contains the following steps:

• **Generation of proton energy spectra library:** FLUKA simulations (version fluka2011.2x.6) [20], [21] were performed to calculate proton energy spectra in different depths of a homogeneous iron target as an approximation of steel alloys. This method is an adaption of the typical setup, by which energy spectra as base data for treatment planning systems in particle therapy are calculated [22]. The spectra were obtained using the USRBDX estimator in one-way scoring mode (to exclude backscattered or backwards emitted protons) at different depths in the phantom. The initial proton energy was set to 220 MeV (typical maximum energy of proton therapy facilities) with an energy spread having a full width at half maximum ΔE/E of 0.1%. The spectra were evaluated in 77 depths from 0 cm (no target) to 1.2· $t_{\mathrm{peak}}$, where $t_{\mathrm{peak}}$ = 42.7 g/cm^2^ is the areal density (hereinafter also referred to as thickness or depth) of the Bragg peak position in iron for a 220 MeV proton beam. All spectra were sorted into the same histogram scheme of 500 equidistant bins from 0 to 250 MeV. A few exemplary spectra are visualized in Fig. 2. Second, the spectra histograms were interpolated in depth such that 2400 histograms were obtained from the originally 77 depths. The interpolation was performed with equidistant fine steps but in the same range as for the 77 steps, meaning that the distance between two spectra histograms corresponds to a thickness of *dt* = 21.35 mg/cm^2^ iron. The discrete spectra are described by:
  (1) $$\begin{array}{cc} & \;{\mathrm{Spc}}_{i,j}=\mathrm{dN}/\mathrm{dE}\left(t_{i},E_{j}\right)\\ & \mathrm{where}\;t_{i}=i\cdot \mathrm{dt}\;\text{;}\;E_{j}=j\cdot 0.5\;\mathrm{MeV} \end{array}$$
  with $t_{i}$ as the *i*-th depth in iron with i=0,…,2400. $E_{j}$ denotes the left border of the *j*-th energy bin with j=0,…,499. In the third step, the spectra were filtered by a porous filter material called LN300 [23]. This foam is a lung substitute with a fine random and porous structure and is usually used for technical quality assurance in radiotherapy. Due to its porosity, it induces a Gaussian modulation of the energy spectra [24]. It is beneficial to irradiate the SPE modulator with such a broader Gaussian energy spectrum compared to a monoenergetic beam because otherwise, the resulting energy spectra would be very sharp in particular at low penetration depths in steel. The Gaussian modulation supports a smoother shape of the low particle contribution at the high-energy side of the (exponentially decreasing) SPE spectra. For the theoretical planning of the SPE modulator we assumed a 14 cm thick LN300 foam block, which has a mean water-equivalent thickness of *t* = 4000 mg/cm^2^ and a modulation power $P_{\mathrm{mod}}$ of 35 mg/cm^2^ (H_2_0) [24]. The modulation power is a quantity used to describe the range smear-out for ions passing porous materials. The characteristic of the LN300 foam block for water corresponds to a mean equivalent thickness in iron of $t_{m}$ = 5760 mg/cm^2^ (Fe) and a modulation power $P_{\mathrm{mod}}$ = 50.4 mg/cm^2^ (Fe), resulting in a particle range sigma of σ = 538.8 mg/cm^2^ (Fe). The foam target is positioned close to the modulator with a distance of 5 cm (see Fig. 5). The broadened proton spectra ${\mathrm{SpcF}}_{i,j}$ in iron are obtained by the following convolution equation:
  (2) $${\mathrm{SpcF}}_{i,j}=\underset{i^{\prime }}{\sum }{\mathrm{Spc}}_{i^{\prime },j}\times C\cdot \exp\left(-\frac{{\left(t_{m}+t_{i}-t_{i^{\prime }}\right)}^{2}}{2\sigma^{2}}\right)$$
  where *C* is an arbitrary normalization constant, the thickness $t_{i}=i\cdot \mathrm{dt}$ and *i* (resp. $i^{\prime }$) is the counter for the different thicknesses for $t_{i}$ (resp. $t_{i^{\prime }}$) with 0⩽i⩽2400. The modulation effect of the foam can be seen in Fig. 2 (dashed line).

**Figure 3:**
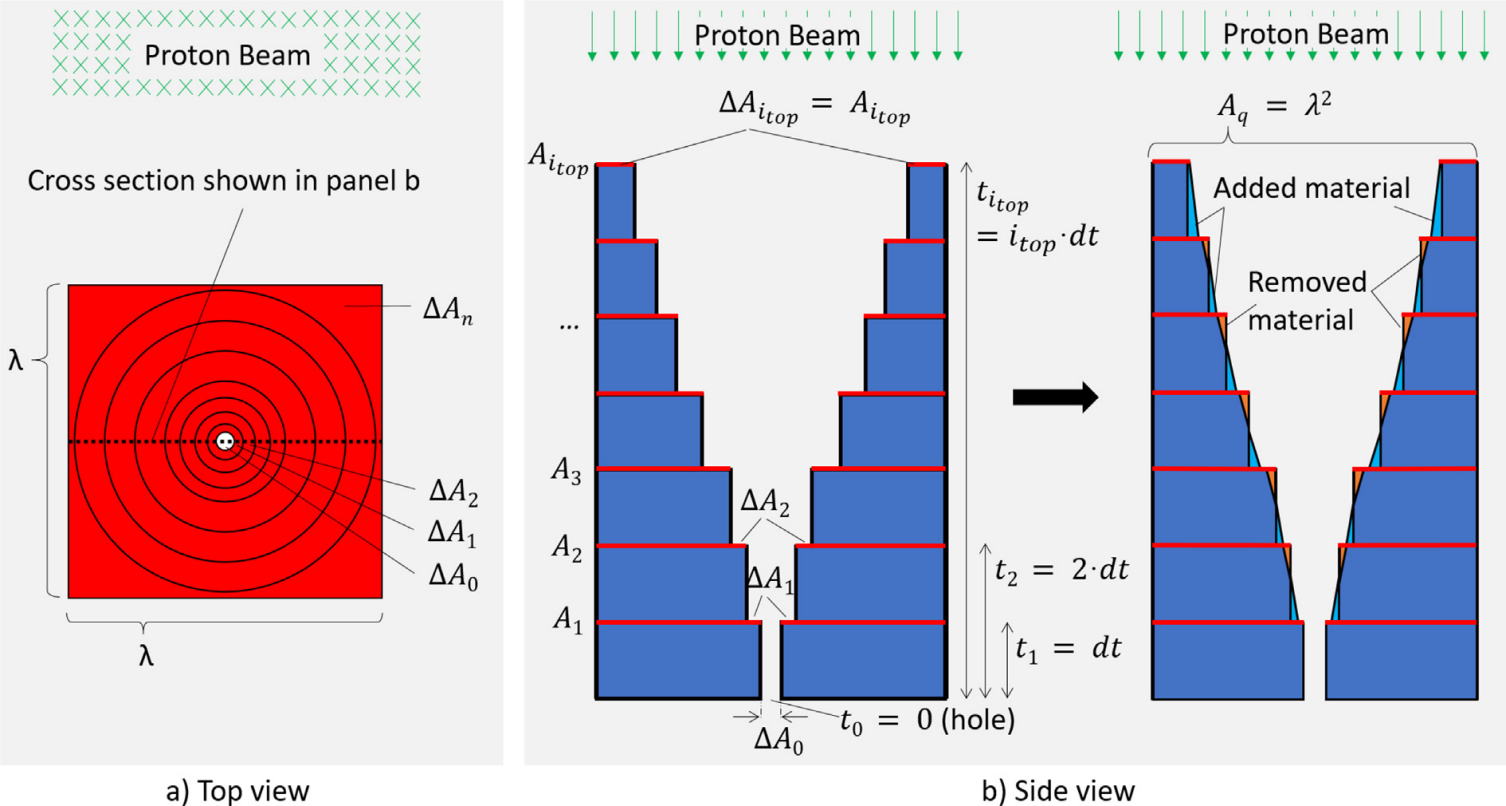
Schematic representation of the structure of the modulator with $i_{\mathrm{top}}$ layers of material with thickness *dt*. a) Top view of the structure with the beam entering the paper plane. b) Side view of the modulating structure with the beam direction from top to bottom. For each layer *i* the area $A_{i}$ orthogonal to the beam direction is smaller than the previous layer ($\Delta A_{n}>0$ and $A_{i}<A_{i-1}$) except for the bottom layer i=0, where this condition does not apply. Each modulator cell has an area of size $\Delta A_{0}$ without any material, which appears as a small hole in the mid of the cell. After the determination of the optimum values of $A_{i}$ (left) the edges of each layer are smoothed by adding/removing material on the layers’ rims (right).

**Figure 4:**
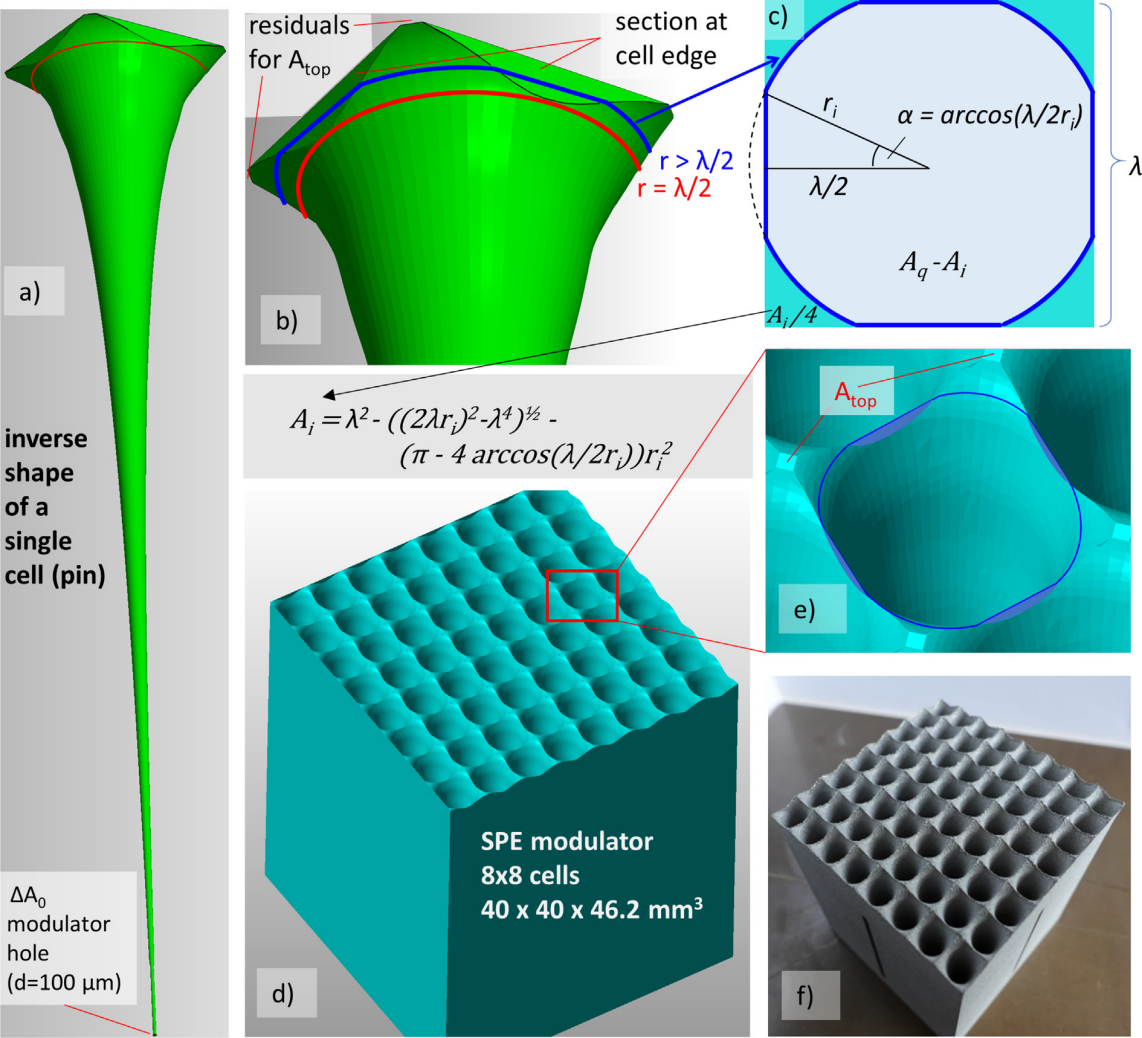
Detailed structure of the modulator. The irradiation direction is from top to bottom in all panels. a) Inverse shape of a single cell, which will be the air-filled volume in the final modulator. At the bottom of the pin, the hole with a diameter d=100 μm is visible. b) Enlargement of the top part of the inverse pin. The highest layer where the circle does not overlap with neighboring cells is represented in red. A higher (larger) layer, where the the circle is cut by the square border of the cell (r>λ/2) is marked in blue. c) Geometry of the pin hole for a layer corresponding to the blue line in b), see also Eq. 6. d) Visualization of the final modulator, compiled of 8x8 cells. The funnel-shaped openings (‘inverse pins’) can be seen from the top. e) Zoom on the funnels opening. The flat residual areas $A_{\mathrm{top}}$ are the areas of maximum material thickness a particle can traverse. f) Photo of the 3D printed steel modulator.
  The advantage of this geometry is, that peak-like structures are also formed at the upper end of the cell, allowing small weights at the bottom and at the top without exceeding the resolution limits of the 3D printer. For instance, a square pyramid shape, as used in [25], of the cell could not be used, because the extremely small weights $w_{0}$ resp. areas $\Delta A_{0}$ of the SPE modulator cannot be realized by any 3D printer for a pyramid shape. Instead, we applied a kind of flipped and inverted circular pyramid (the flipped pyramid represents the open volume of the cell, like a funnel).
  Finally, $t_{i}$ was scaled in beam direction by the steel density 7.9 g/cm^3^ and together with the layers $A_{i}$ the 3D-contour of modulator structure can be exported as a stl-file for printer input. It should be noted that for the concrete calculation of the funnel shape, $r_{i}$ must be calculated as a function of $A_{i}$, which is the inverse function of Eq. 6, which cannot be determined analytically. In this respect $r_{i}$ must be calculated numerically from Eq. 6 (e.g. by Newton’s method).
  Fig. 4 shows the result of the optimization and design process. Fig. 4a and b show the inverse (open) volume of the funnel and also depict the extremely small opening $\Delta A_{0}$. Fig. 4c, d and e show the modulator cell resp. cell array, visualize the transition at r>λ/2, where the circular funnel touches the cell border and depicts the geometry of Eq. 6. A photo of the 3D-printed modulator is shown in Fig. 4f with a period λ=5mm of the cells, which allows a good trade-off between a not too filigree aspect ratio of the structures (too thin holes cannot be printed) and a not too long distance to the irradiated target concerning the mandatory blur-out of the lateral dose inhomogeneities (see above and Fig. 7). Even tough the mathematical optimization would deliver a thinner hole, we set a minimum limit of 100 μm for the hole diameter $\left(2r_{0}\right)$ considering the printing limitations and taking into account that the beam has a certain divergence (at least 13 mrad from scattering at the foam) and cannot pass properly a too narrow hole.
• **Manufacturing of the modulator:**
  Due to its high density, the usage of steel enables compact modulator dimensions compared to 3D-printing with polymers. The strongly reduced size (factor ≈5) of the structures in beam direction reduces the gradients of its flanks and length of the narrow funnels and thus significantly facilitates the printing process. A further advantage is, that the extremely narrow end of the funnel is much shorter (compared to polymers), therefore slightly scattered particles can easier pass (in a more defined way) the hole. Recent developments in 3D laser printing technology allow meanwhile to print steel objects with a precision of typically 20–50 μm resolution [26], which is comparable to the precision of polymer 3D-printing systems (e.g. stereo lithography laser 3D printer). Finally, steel also ensures a good mechanical robustness, even for fine structures and shows - compared to polymers - no aging effect, even for higher beam intensities/doses.
  A structure of 8 × 8 modulating cells with a design as described before was 3D-printed using the ‘selective laser melting’ (SLM) technique. A ‘TruPrint 1000’ (company Trumpf, Ditzingen, Germany) machine produced the modulator in stainless steel (material designation: ‘1.4404’, 17% Cr, 12% Ni and 2.0% Mo, density: 7.9 g/cm^3^). This modulator has a height of 46.21 mm and a lateral size of 40 × 40 mm^2^. The most critical structures are the holes at the bottom of the funnels with a diameter of 100 μm, which was the minimum diameter of an opening that could be produced with this technique. It should be noted that the lateral extension of this modulator is relatively small, because it was designed only for physical testing and not for radio-biological irradiations of larger samples. 3D printing of larger modulators is unproblematic, but the printing costs increase. The SLM 3D-printing of the presented modulator cost about 2000 €.

### 2.2 Experimental setup for modulator verification

**Figure 5:**
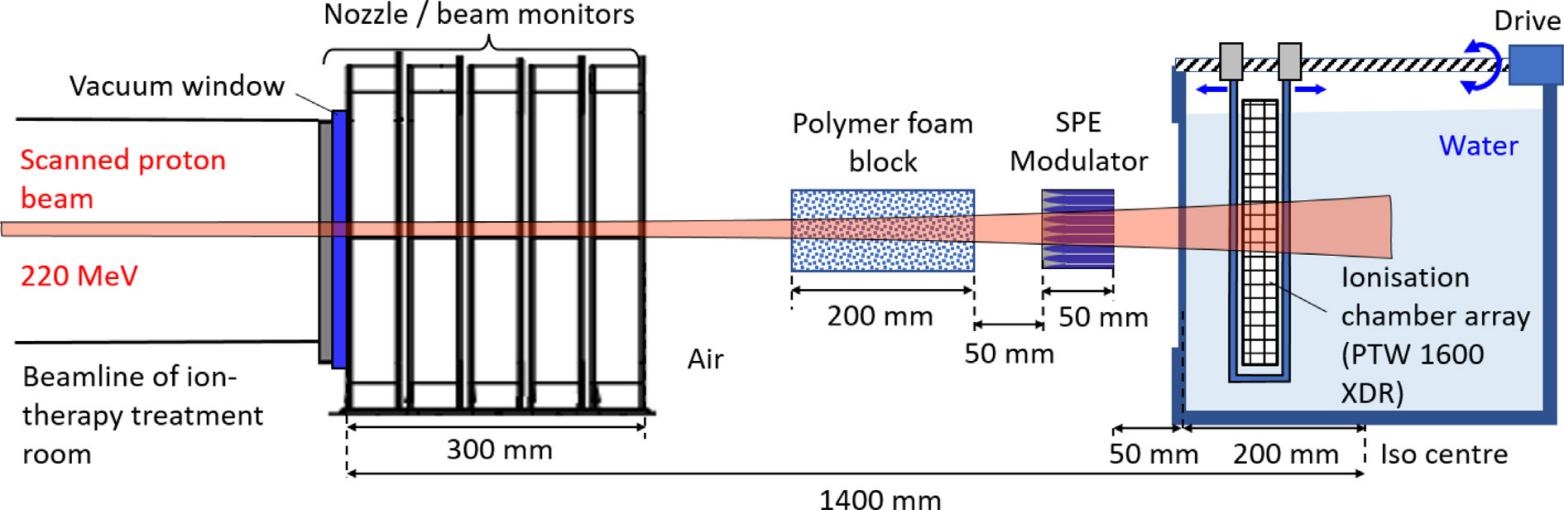
Sketch of the measurement setup for the depth-dose-profile downstream the SPE modulator (not to scale). The scanned 220 MeV proton beam exits from the vacuum beamline through a foil window. After passing the Nozzle the beam is filtered by a 20 cm polymer foam block to broaden the beam energy. The dose profile generated by the SPE modulator is measured in a large water tank, in which an ionization chamber array *PTW OCTAVIUS 1600XDR* is placed. By changing the depth position of the array a DDP can be acquired. The size of the scanned primary proton field was 9 × 9 mm^2^.

In order to ensure that design and manufacturing of the SPE modulator were executed as planned, a measurement of the depth-dose profile (DDP) from the field emitted downstream of the modulator was performed at the Marburg Ion Therapy Center (MIT). The kinetic energy of 220 MeV is in the high-energy limit of the ion synchrotron in Marburg and was selected in agreement with the theoretical planning of the modulator. The proton beam was scanned over the target in a field of 9 × 9 mm^2^ size with 3 mm spacing and 9.5·$10^{8}$ particles per spot. The experimental setup is visualized in Fig. 5. First, the proton beam leaves the beam line through the exit window and the beam monitors. A set of four 5 cm thick polymer Gammex LN-300 foam blocks [23] is applied to broaden the sharp peak of the proton energy spectrum before the beam enters the SPE modulator (positioned with an angular accuracy <1 mrad). This has a similar effect to a ripple filter as applied in [18], but - due to fine porous structure - allows a smaller distance to the modulator. It should be noted, that this LN300 foam block has a slightly larger thickness, than the foam blocks theoretically assumed in the design process (5760 mg/cm^2^ (H_2_0) instead of 4000 mg/cm^2^ (H_2_0)), but on the other hand a lower modulation power (21 mg/cm^2^ instead of 35 mg/cm^2^), so that the modulation effect is similar. Finally, the beam penetrates a large water tank [27] in which the radiation detector is placed. The ionization chamber array *PTW OCTAVIUS 1600XDR* was chosen since it not only allows the measurement of the laterally-integrated DDP but also acquires the lateral distribution of dose. The detector consists of a 15 × 15 cm^2^ matrix with 1521 cubic ion chambers (each 2.5 × 2.5 × 2 mm^3^) with center-to-center spacing of 2.5 mm in the central area and 5 mm in the surrounding area. The effective water-equivalent depth of measurement in the OCTAVIUS is 6.9 mm below the surface, which is made of polystyrene.

### 2.3 Monte Carlo simulations and comparisons

*FLUKA for spectra library for optimization.* The Monte Carlo particle transport code FLUKA was used to simulate the library of proton kinetic energy spectra after passing selected material thicknesses of iron. The details are provided in subsection 2.1, see first step in the modulator development.

*Geant4 simulations.* Geant4 Monte Carlo simulations (version 11.0.0) [28], [29], [30] were performed to investigate the radiation field produced by the SPE modulator and to compare the results to measurement data. The physics interactions were processed by the physics processes included in the pre-compiled QGSP_BIC_EMY reference physics list. The included Urban scattering model is compatible with Geant4-based standard tools used in space radiation research like SPENVIS/GRAS. Two different scenarios were simulated to investigate (i) the obtained SPE kinetic energy spectrum, (ii) the produced 2D-fluency distribution, and (iii) the laterally-integrated DDP behind the modulator:

• Simulation of proton and neutron energy spectra and 2D fluence distribution in air behind the modulator: The beam exit window was modeled as a 0.254 mm thick Kapton foil followed by a replacement for the complex beam monitor system, which was simulated as a water block of 30 × 30 × 30 cm^3^ with a density of 1/270.3 g/cm^3^ equivalent to 1.1 mm of water. For the planning of the modulator and during the experiment, a polymer foam block was used to broaden the energy distribution of the sharp primary beam. This was modeled through an adjusted primary beam energy of 202.5 MeV and an energy sigma of $\sigma_{E}$=1.65 MeV, which corresponds to the modulation by the foam used in the modulator optimization process. The beam was scanned over an area of 40 × 40 mm^2^ to cover the full modulator with a Gaussian spatial profile with FWHM = 8.1 mm. The kinetic energy spectra were scored in a 20 × 20 × 0.1 mm^3^ cuboid volume 1 mm downstream of the SPE modulator. The 2D fluence distribution was scored in an air-filled box of 5 × 5 × 20 cm^3^ with 1 bin in x-direction, 500 bins in y-direction and 2000 bins in z-direction. The start of the box was positioned 2.5 cm upstream the modulator in order to score the fluence not only behind the modulator but also anterior and lateral to it. Note that the geometry is symmetric for x and y. The simulation was performed with 2.5·10^7^ primary particles.
• Simulation of the depth dose distribution in water behind the modulator: The experiment described in subsection 2.2 was re-simulated. The OCTAVIUS detector is modeled as a polystyrene box of 30 × 30 × 2.2 cm^3^ with a laterally-centered, air-filled active volume of 15 × 15 × 0.2 cm^3^ following 6.9 mm polystyrene, which covers the active volume. All other length scales and the projectile scanning field are replicated according to the description in subsection 2.2. In total, 5$\cdot 10^{6}$ primaries were simulated per detector depth in the water target. Within the active volume, the dose is scored in ionization chamber units of 2.5 × 2.5 × 2 mm^3^ in accordance with the specifications of the OCTAVIUS detector. Since the realistic description of foam materials in Monte Carlo codes is complicated, the LN-300 foam was modeled as a water box of the size of the foam (10 × 10 × 20 cm^3^) with a reduced density of ρ=0.288 g/cm^3^ to match the mass of the foam used in the experiment. The primary proton beam energy was set to E=220.0 MeV with an energy sigma $\sigma_{E}$=1.58 MeV to mimic the range smear-out by the LN-300 foam used in the experiment. In agreement with the experimental setup, the primary beam was scanned over an area of 9 × 9 mm^2^, with a Gaussian spatial profile with FWHM = 8.1 mm. In order to be conform with the size of the scorer for the SPE spectrum, the DDP was evaluated in the centered detector cells covering 20 × 20 mm^2^.

The uncommon use of two different Monte Carlo codes for separate parts of a single project was chosen to keep the optimization process and the used software comparable to the ones typically used for the optimization of medical modulators (FLUKA)[12], whereas the Geant4 simulations were later employed for simulations with heavier ions and high geometrical complexity and are the GSI standard for all tools developed for the GCR simulator [10].

## 3 Results

**Figure 6:**
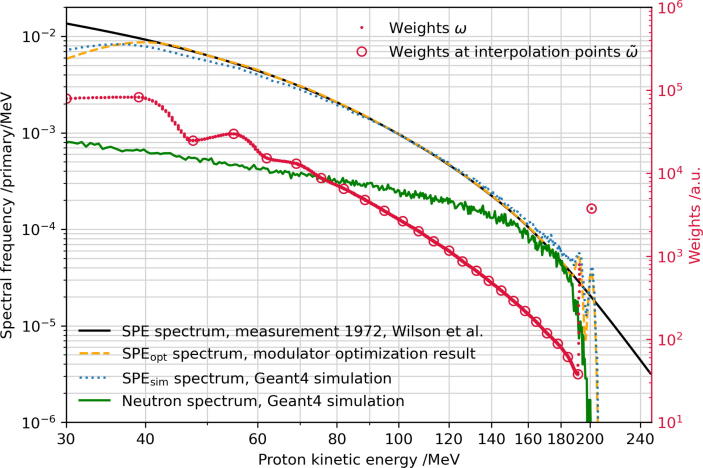
SPE energy spectra as measured in August 1972 [16], as obtained as a result of the modulator shape optimization and as simulated with the Monte Carlo radiation transport code Geant4 (left y-axis); green line shows the GEANT4 neutron spectrum behind the modulator. Furthermore, the weights of the designed modulator are shown (right y-axis). The x-coordinates of the data points for the weights $w_{i}$ in this plot correspond to the energy peak position of the corresponding spectra ${\mathrm{SpcF}}_{i,j}$ in the depth $t_{i}$ (see Eqs. (1), (2)).

Fig. 6 shows the kinetic energy spectrum produced by the described SPE modulator. The theoretically expected spectrum ${\mathrm{SPE}}_{\mathrm{opt}}$ according to the optimized weights (see Eq. (4)) matches the measured SPE spectrum used during the optimization process over the full intended energy range from 40 to 200 MeV. An exception to this is found at high proton energies, where oscillations are visible in the modulated spectrum. This part of the spectrum is formed by protons passing thin or zero material thicknesses as present at the end of the pin-shaped holes in the modulator. Due to manufacturing limitations, the last part of the pin-shaped hole is formed cylinder-like leading to sharp material edges, which result in the expected oscillations of the SPE spectrum at high proton energies. However, the contributions of these high proton energies to the full SPE spectrum are relatively small. In contrast to the analytical modulator optimization technique, in the Geant4 Monte Carlo simulations, the physical interactions of the primary beam with the modulator are explicitly simulated, such as scattering and target fragment production. At lower proton energies (≈40–100 MeV), the proton yield is slightly smaller compared to the (1D) optimized spectrum due to the increased scattering for lower-energetic particles. It should be noted that the FLUKA spectra and the modulator optimization (see Eq. (3)) only consider the longitudinal coordinate of the beam and, thus, do not include scattering explicitly.

At high proton energies the Geant4 simulation leads to a slightly increased proton yield and reproduces the above-described oscillations in the SPE spectrum. The optimized and simulated spectra agree concerning the maximum proton energy. The simulated spectral neutron fluence is one order of magnitude smaller than the proton spectrum, only in the high energy range (140–190 MeV) - where the fluences are low anyway - neutrons reach ≈50% of the protons fluence.

**Figure 7:**
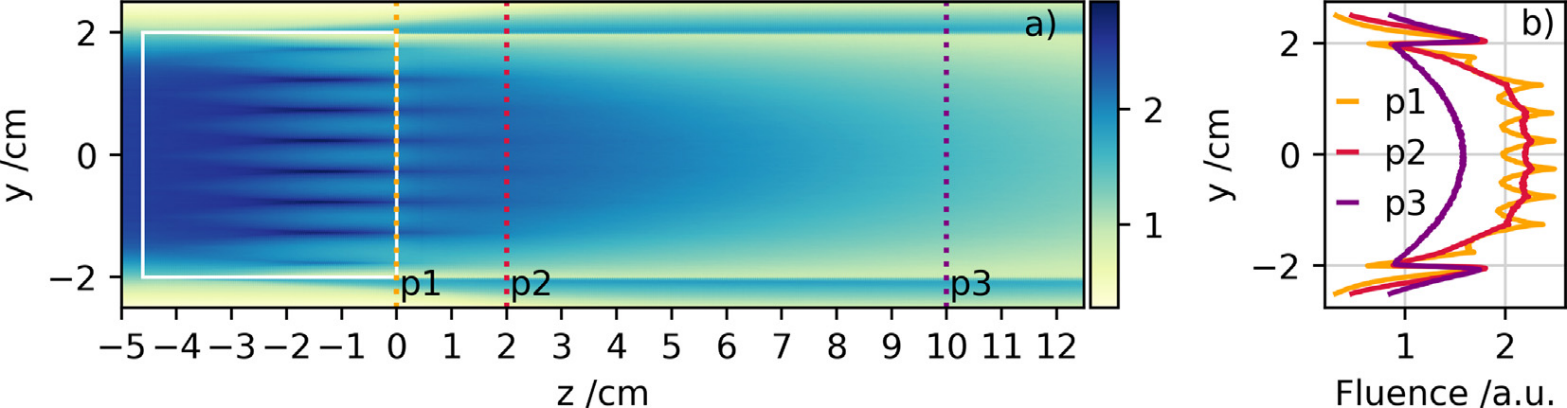
Geant4 simulation of the fluence distribution surrounding the irradiated SPE modulator. a) 2D fluence distribution upstream, within, and downstream the modulator. The modulator is surrounded by air and the position of the modulator is indicated as a white box. The color bar defines the fluence in arbitrary units and the z-directions is set as the primary beam direction. The three colored, dotted vertical lines correspond to the slices parallel to the y-axis for which the 1D fluence distributions are shown in panel b).

In experimental applications of the SPE modulator, a homogeneous irradiation field at the target position is essential to realistically reproduce space radiation scenarios. The simulation of the 2D fluence distribution behind the modulator allows to determine the optimum distance downstream sufficient for target positioning. As visualized in Fig. 7, the periodic structure of the inverse pins in the modulator are still visible within the first few centimeters behind the modulator before the structure smears out after approximately 5–10 cm. In this simulation scenario, the modulator is irradiated with a primary field of the size of the modulator (40 × 40 mm^2^). Thus, undesirable effects at the edges of the modulator are visible. Depending on the size of the target to be irradiated with SPE radiation, the size of the modulator and the irradiation field will be increased.

**Figure 8:**
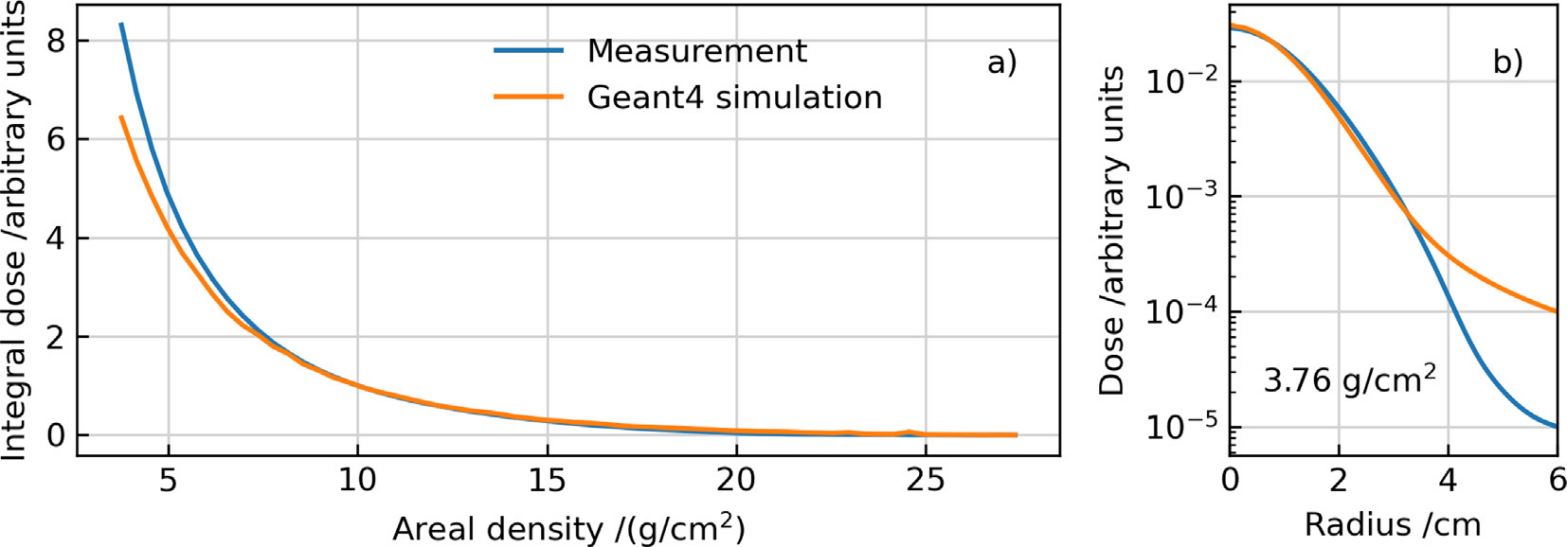
Measured and simulated DDP downstream the SPE modulator (a). The corresponding experimental setup is shown in Fig. 5. This experiment and simulation were performed by scanning a 9 mm × 9 mm primary proton radiation field. The areal density includes all material beyond the modulator including the PMMA walls of the water tank and the detector holder, the polystyrene of the OCTAVIUS detector in front of the active area, and water. The dose curves are normalized at an areal density of 10 g/cm^2^ and the DDP in panel (a) is integrated over the central lateral scoring area of 20 × 20 mm^2^ for both, the measurement and simulation. The dose within the beam profile (b) is shown as a function of the radius exemplary at a depth in the water tank corresponding to an areal density of 3.76 g/cm^2^.

To further validate the shape and functionality of the printed modulator, the laterally-integrated DDP was measured in water according to the setup described in the methods section. As depicted in Fig. 8 the dose decreases roughly exponentially with depth in water. The Geant4 simulations reproduce the general shape of the measured DDP. However, at intermediate material thicknesses, a slight underestimation of the dose is observed. Closer investigations indicate that this is due to an increased scattering in the simulations, leading to a dose halo, that is stronger than for the experimental data (compare Fig. 8b). Geant4 benchmarking studies showed an overestimation of the beam halo [31]. Additionally, no benchmarking exists for steel so far. Thus, inaccuracies in the scattering model cannot be excluded.

## 4 Discussion

This work serves as a general ”recipe” for the planning, manufacturing and testing of SPE modulators and can be applied to any other SPE spectrum. Following this proof-of-concept, the next generation of modulators will incorporate a larger base area in order to be able to irradiate samples with a spatial extension of more than a few centimeters homogeneously. In the typical application example of the SPE modulator a scanned beam will be used to irradiate the modulator with protons with a dose rate typical for therapy applications. However, the SPE modulator can be used with passively enlarged fields or even single pencil beams if the lateral size of the primary proton beam is large enough in relation to the period λ of the modulator structure. If the field size is too small or the modulator cells are too large, deviations of the produced SPE spectrum compared to the planned SPE spectrum will be observed. In the experiment described in this work for example a pencil beam with FWHM = 8.1 mm was used to irradiate the modulator with λ=5 mm. Thus, the lateral oscillation was smaller than 0.1% (See supplementary material of [32] for details.).

The discrepancies between the measurement and the MC simulation (Fig. 8b) indicate an uncertainty in the large-angle halo of the modulated (scattered) field. Further measurements and adjustments to the MC code would be desirable to better understand the origin of the observed deviations. Detailed testing and analysis of the influence of different Geant4 scattering models on the results might provide further insights. However, the measured lateral distribution is more reliable than the simulation and a lower halo provides an advantageous effect for the modulator. If less particles are scattered out at the border of the field a more homogeneous radiation field is obtained. In general, the scattering problem of the modulator can always be solved by enlarging the modulator and the scanned field because this results in a larger homogeneous radiation field. In contrast to that the spatial extend of the beam halo remains the same. However, the larger modulator and scanning field come at the price of higher printing costs and more required beam time. The validation of the SPE modulator via the measurement of the depth dose distribution is in the opinion of the authors the best method from a practical point of view. A direct precise measurement of the proton spectrum in the range of 40–220 MeV is technically possible but means high effort and sophisticated tuning and calibration of the setup requiring a lot of beam time. Since protons in the relevant energy range  > 40 MeV are low-LET particles, their contribution to the total physical and biological effective dose is similar per proton. Thus, the total dose (instead of the underlying proton energy spectra) is the most relevant parameter for biological and electronic radiation hardness tests. The depth dose test in combination with MC simulation enables an assessment also of the delivered spectrum because of the unique relation of the DDP and the energy spectrum: One can imagine the depth dose profile as the superposition of monoenergetic Bragg curves and their weights correspond to the energy spectrum. Furthermore, from the longitudinal extend and shape of the DDP, one can draw conclusions about the the energy spectrum of the present particle field. This can also serve as a internal benchmark. For the presented modulator, a good agreement of the simulated spectrum with the initial SPE spectrum of 1972 can be observed with a deviation in the order of 10% for the lower energy part. The deviation (oscillation) at the high energy end of the spectrum is not very relevant, because the contributions are generally low and oscillations average out themselves.

The modulation performance of the SPE modulator is not directly linked to the intensity of the primary proton field and therefore the presented modulator can be used at any beam intensity. However, for statistical reasons, SPE irradiations require a certain amount of primary particles for a reasonable approximation of the desired energy distribution. To blur out the geometrical structure of the modulator and spatially homogenize the kinetic energy of the radiation field, a suitable air gap of 10–20 cm between the modulator and the target is recommended [19]. Additionally, this air gap mitigates the impact of low-energy target fragments produced in the modulator material. It is important to note, that the use of passive modulators will create additional neutrons and is not producing a pure proton field, which should be considered when exposing cells or electronic components to a passively modulated radiation. However, while basically all impinging protons deposit energy inside the irradiated object, most of the neutrons will penetrate through it without interaction. Therefore, at least for absorbed dose, the additional neutron contribution is of minor relevance and neutron fluence would be dominated by neutrons generated in an additional shielding target behind the modulator (which is a likely application scenario for the SPE modulator).

Extreme cases with a rather flat SPE spectrum up to kinetic energies of 1 GeV as measured in 1956 [16] or calculated worst-case SPEs [33] might lead to very fragile or outright impossible-to-manufacture modulating structures. Therefore, in particular for such extreme SPEs, it could be beneficial (but also more expensive) to use printing materials with a larger density than steel for the modulator such as tungsten. Alternatively, the full SPE irradiation could be subdivided into several modulators and primary beam energy combinations similar to the hybrid active–passive simulation approach as used for ground-based GCR simulation [10]. This allows the modulation of high proton energies, while at the same time, a compact and stable structure of the modulator is ensured. However, it needs to be confirmed that these high-density modulators are producible with available 3D printing techniques.

Furthermore, for future modulators, we recommend including the distance between the modulator and the irradiation target in the optimization process. Therefore, the generation of 3D Monte Carlo base data is needed, which includes the desired target dimensions and the aforementioned distance to the target. This reduces deviations in the energy spectrum induced by scattering processes, especially for lower-energetic protons. Additionally, to irradiate samples with a size exceeding 20 × 20 mm^2^ it is required to print a modulator with a larger lateral extension (>40 × 40 mm^2^) to achieve a homogeneous dose distribution and energy spectrum covering the whole target.

As mentioned in the introduction, the follow-up project of the SPE modulator, still under development, is the GCR modulator [10]. The concept and design of these modulators will be similar, even though they will be partially printed as polymer filters (in order to produce more lighter fragments from the ${}^{56}$Fe beam) and are therefore more stretched. In general, GCR-like radiation is difficult to generate with only one filter and one accelerator energy, therefore three different energies and modulators are needed and will be sequentially applied. The much more challenging task (compared to a SPE modulator) is to match the energy spectra not only for one species (protons) but for many other species between protons and iron. In addition, the strongly scattered light fragments make the optimization problem necessarily three-dimensional, while the only one-dimensional optimization for the SPE modulator already achieves satisfying results.

## 5 Conclusion

This work describes the shape optimization, manufacturing, and testing of a passive 3D-printed SPE modulator. By irradiating the modulator with monoenergetic protons, as available at many research or clinical accelerators, the beam is converted instantaneously into the proton energy spectrum of the SPE measured in August 1972. While this work serves as a proof of concept, the described method is easily transferable to other SPE spectra. As of now, the radiation modulator is available to the research community performing experiments in corresponding space radiation scenarios. It enables an efficient and fast exposure of biological or electronic samples.

## Declaration of Competing Interest

The authors declare that they have no known competing financial interests or personal relationships that could have appeared to influence the work reported in this paper.
